# Inhibition of JAK1/2 Tyrosine Kinases Reduces Neurogenic Heterotopic Ossification After Spinal Cord Injury

**DOI:** 10.3389/fimmu.2019.00377

**Published:** 2019-03-07

**Authors:** Kylie A. Alexander, Hsu-Wen Tseng, Whitney Fleming, Beulah Jose, Marjorie Salga, Irina Kulina, Susan M. Millard, Allison R. Pettit, François Genêt, Jean-Pierre Levesque

**Affiliations:** ^1^Mater Research Institute – The University of Queensland, Translational Research Institute, Woolloongabba, QLD, Australia; ^2^CIC-IT 1429, Service de Médecine Physique et de Réadaptation, Raymond Poincaré University Hospital, AP-HP, Garches, France; ^3^Université de Versailles Saint Quentin en Yvelines, END:ICAP Inserm U1179, Montigny le Bretonneux, France

**Keywords:** spinal cord injury complications, heterotopic ossification, JAK- STAT signaling pathway, ruxolitinib, oncostatin M receptor

## Abstract

Neurogenic heterotopic ossifications (NHO) are very incapacitating complications of traumatic brain and spinal cord injuries (SCI) which manifest as abnormal formation of bone tissue in periarticular muscles. NHO are debilitating as they cause pain, partial or total joint ankylosis and vascular and nerve compression. NHO pathogenesis is unknown and the only effective treatment remains surgical resection, however once resected, NHO can re-occur. To further understand NHO pathogenesis, we developed the first animal model of NHO following SCI in genetically unmodified mice, which mimics most clinical features of NHO in patients. We have previously shown that the combination of (1) a central nervous system lesion (SCI) and (2) muscular damage (via an intramuscular injection of cardiotoxin) is required for NHO development. Furthermore, macrophages within the injured muscle play a critical role in driving NHO pathogenesis. More recently we demonstrated that macrophage-derived oncostatin M (OSM) is a key mediator of both human and mouse NHO. We now report that inflammatory monocytes infiltrate the injured muscles of SCI mice developing NHO at significantly higher levels compared to mice without SCI. Muscle infiltrating monocytes and neutrophils expressed OSM whereas mouse muscle satellite and interstitial cell expressed the OSM receptor (OSMR). *In vitro* recombinant mouse OSM induced tyrosine phosphorylation of the transcription factor STAT3, a downstream target of OSMR:gp130 signaling in muscle progenitor cells. As STAT3 is tyrosine phosphorylated by JAK1/2 tyrosine kinases downstream of OSMR:gp130, we demonstrated that the JAK1/2 tyrosine kinase inhibitor ruxolitinib blocked OSM driven STAT3 tyrosine phosphorylation in mouse muscle progenitor cells. We further demonstrated *in vivo* that STAT3 tyrosine phosphorylation was not only significantly higher but persisted for a longer duration in injured muscles of SCI mice developing NHO compared to mice with muscle injury without SCI. Finally, administration of ruxolitinib for 7 days post-surgery significantly reduced STAT3 phosphorylation in injured muscles *in vivo* as well as NHO volume at all analyzed time-points up to 3 weeks post-surgery. Our results identify the JAK/STAT3 signaling pathway as a potential therapeutic target to reduce NHO development following SCI.

## Introduction

Neurogenic heterotopic ossification (NHO) is the abnormal formation of extra-skeletal bones in muscles (1), mostly periarticular (2), and is a frequent and very incapacitating complication in patients with spinal cord injury (SCI) (15–25%) and traumatic brain injuries (5–12%) (3, 4). NHO prevalence is higher in combat-inflicted trauma particularly in victims of explosive blasts with associated SCI or TBI where NHO prevalence is over 60% (5, 6). NHOs are debilitating due to their size (up to 2 kg), causing significant pain and gradual reduction in the range of motion of affected limbs, often progressing to complete joint ankylosis. This exacerbates functional disabilities by increasing difficulty in sitting, eating and dressing (7). NHO growth can also cause nerve, blood vessel compression, and irreversibly damage the affected joint further increasing patient morbidity (8). Despite knowing this pathology for 100 years, treatment is currently limited to surgical resection after NHO have matured and become symptomatic (2, 7, 9–11). The surgical procedure is challenging, particularly when ossifications entrap joints, large blood vessels and nerves. Furthermore, even after resection, NHO recurrence is observed in at least 6% patients (1, 2, 9, 12). The development of improved treatments for NHO has been slow and trials of pharmacological interventions have continued to show limited effectiveness, reflecting the current limited knowledge on the etiology and pathophysiology of NHO. Identification of therapeutic targets to block NHO development in SCI/TBI patients remains a priority in order to decrease the prevalence and morbidity of this pathology (5).

We have developed the first clinically relevant animal model of NHO following SCI in genetically unmodified mice (13). In this model the combination of two injuries is necessary to trigger NHO development in the muscle: a severe lesion of the central nervous system such as a SCI in combination with a muscle injury (13). Development of NHO following spinal cord transection in this model was triggered by macrophages infiltrating damaged muscles exclusively in the context of a complete SCI (13, 14). More recently we established that the inflammatory cytokine oncostatin M (OSM) secreted in part by macrophages infiltrating the inflamed muscle contribute to both human and mouse NHO development (15). This is consistent with the pleiotropic role of OSM in regulating skeletal bone formation and resorption (16, 17) and the previous demonstration that macrophage-derived OSM can promote mesenchymal stem cell osteogenic differentiation (18) and intramembranous bone formation (19). OSM was shown to be elevated in the serum of patients developing NHO and OSM produced by activated macrophages isolated from NHO biopsies promoted osteoblastic differentiation and mineralization of human muscle-derived stromal cells extracted from NHOs (15). Likewise in mice, SCI caused the abnormal and persistent expression of OSM in the injured muscles. Importantly, mice defective for the OSM receptor (OSMR) α chain gene *Osmr* had significantly reduced NHO volumes in response to SCI and muscle injury (15). Overall our results provide strong evidence that macrophages contribute to NHO formation in part through the osteogenic action of OSM on muscle cells suggesting that OSM/OSMR signaling could be a suitable therapeutic target for NHO.

OSM is a member of the interleukin (IL)-6 family of cytokines which include IL-6, IL-11, leukemia inhibitory factor (LIF), cardiotrophin-1, and ciliary neurotrophic factor. These cytokines bind to diverse heteromeric receptors with a common glycoprotein 130 (Gp130) chain. Binding of IL-6 family cytokines to their cognate receptors, all of which comprise a common gp130 subunit, causes the activation of Janus tyrosine kinase (JAK)-1 and JAK2 which in turn tyrosine phosphorylate signal transducer and activator of transcription (STAT)-1 and STAT3 (20, 21). Once tyrosine phosphorylated (p), pSTAT1, and pSTAT3 translocate to the nucleus and activate the transcription of a large array of genes depending on the cell type. Mouse OSM binds with a strong affinity to the OSMR:gp130 complex and with a 30-fold lower affinity to the leukemia inhibitory factor receptor (LIFR):gp130 complex (22). Typically, OSM binding to the OSMR:gp130 complex causes the phosphorylation and activation of both STAT1 and STAT3 via JAK1/2 (23, 24) which in turns leads the transcription of a large range of genes that include suppressor of cytokine signaling (SOCS)-3. A negative feed-back loop is triggered by SOCS3, which binds to both gp130 and activated JAKs, suppressing this signaling cascade and STAT1 and STAT3 activation (25, 26).

Since OSM and OSMR play an important role in NHO pathogenesis following SCI (15), we further examined STAT3 activation status in mouse muscles during NHO development. We confirmed that muscle satellite and interstitial cells isolated from mouse muscles express OSMR, with JAK1/2-dependant tyrosine phosphorylation of STAT3 in response to OSM. In addition, we found higher and persistent STAT3 tyrosine phosphorylation in injured muscles of SCI mice developing NHO. We show that this persistent STAT3 phosphorylation and activation in the injured muscle is an important driver of NHO as administration of ruxolitinib, a small synthetic inhibitor of JAK1/2 tyrosine kinases used to treat myelofibrosis and polycythemia vera caused by activating mutations of JAK2 (27, 28), significantly reduced STAT3 phosphorylation in the injured muscles of mice. Importantly, ruxolitinib administration also significantly reduced NHO development following SCI.

## Materials and Methods

### Animals

C57BL/6 mice were obtained from Animal Resource Center (Perth, Australia). All mouse procedures were approved by the Health Sciences Animal Ethics Committee of The University of Queensland and performed in accordance with the Australian Code of Practice for the Care and Use of Animals for Scientific Purposes.

### NHO Mouse Model

NHO mouse model was carried out as previously described (15) by performing a spinal cord transection between T11 and T13 together with intramuscular injection (i.m.) of cardiotoxin (CDTX) purified from the venom of Naja pallida (Latoxan) at 0.32 mg/kg in the hamstring muscles under general anesthesia (100 mg/kg Ketamine, 10 mg/kg xylazine, and 1% isofluorane). Control mice underwent sham-surgery and/or intramuscular injection of equal volume of phosphate buffered saline (PBS). In this model, NHO develop in the CDTX-injected muscle within 1–3 weeks (13, 15). Post-surgery, mice were administered ruxolitinib phosphate (LC Laboratories) 60 mg/kg by oral gavage twice daily from day 0 to day 7 post-surgery. Ruxolitinib phosphate powder was first dissolved as a 4X stock in dimethyl sulfoxide (DMSO) then diluted to 1X in vehicle (5 mg/ml hydroxypropyl methylcellulose, 0.1% Tween 20 in water). Control mice were gavaged with 25% DMSO in vehicle.

### Tissue Collection

At 1–3 weeks post-surgery mice were euthanized by CO_2_ asphyxiation. For histological analysis, the hind limbs were fixed in PBS with 4% paraformaldehyde as previously described (15). For western blots, muscle samples were harvested at specified time points and immediately placed in ice-cold protein extraction buffer (300 mM NaCl, 30 mM Tris-HCl, 1% Triton-X 100) containing a house made cocktail of phosphatase inhibitors (10 mM EDTA, 0.1% NaN_3_, 20 mM NaF, 1 mM Na_3_VO_4_, 10 mM β-glycerophosphate, 10 mM levamisole) buffered at pH 7.4 and supplemented with 1X protease inhibitor cocktail (Complete™ ULTRA Tablets, Roche) and snap frozen in liquid nitrogen until extraction.

### Muscle Cell Isolation, Sorting, and Culture

Isolation, sorting and culture of muscle CD45^−^Ter119^−^CD31^−^CD34^+^Sca1^−^ satellite cells and CD45^−^Ter119^−^CD31^−^CD34^+^Sca1^+^ interstitial cells was carried out as previously described (15). For pSTAT3 phos-flow analysis, cultured muscle satellite cells, interstitial cells, and the mouse mesenchymal progenitor cell line Kusa4b10 cells (29, 30) were detached by incubating cell monolayers in PBS plus 4 mM EDTA for 5 min at 37°C. Once in suspension, cells were washed in Dulbecco modified essential medium (DMEM, Gibco, Life Technologies), centrifuged and resuspended in DMEM. Cell aliquots (1 × 10^6^) were then preincubated with or without 1 μM ruxolitinib (LC Laboratories), for 30 min at 37°C and subsequently stimulated by addition of 25 ng/mL recombinant mouse OSM (R&D Systems) for 10 min. Cells were then immediately washed in 10 mL ice cold Tris-buffered saline pH 7.4 containing 1 mM Na_3_VO_4_, centrifuged, and cell pellets resuspended for fixation and permeabilization (BD Cytofix, Perm buffer IV, BD Biosciences) for 10 and 30 min respectively, cells were then stained with AlexaFluor647-conjugated mouse anti-pSTAT3 (pY705) monoclonal antibody (BD Biosciences, catalog # 557815) for 30 min on ice, cells were then washed and subsequently run on a LSR Fortessa x20 flow cytometer (BD Biosciences). Files were subsequently analyzed with Flow Jo software version 10.4.

### Protein Extraction and Western Blot

Frozen muscle samples were thawed and homogenized using a TissueRuptor (Qiagen), in ice cold protein extraction buffer for 3 rounds of 20 s at top speed, incubated on a horizontal rotator at 4°C for 15 min. Tissue debris were removed by centrifugation at 15,000 g at 4°C for 20 min. Supernatants were taken and protein concentrations measured using a BCA assay (ThermoFisher). Muscle lysates (25 μg protein per lane, one separate mouse per lane) were loaded on a 4–12% acrylamide Bis-Tris pre-cast gel (ThermoFisher), and subsequently wet transferred onto a Hybond C Extra membrane (Amersham Biosciences) and blocked with Odyssey Blocking Buffer. Primary antibodies included an anti-total-STAT3 rabbit monoclonal antibody (mAb) clone 79D7 diluted at 1/2,000 (Cell Signaling), anti-phospho-STAT3 (Tyr705) XP® rabbit mAb clone D3A7 (Cell Signaling) diluted 1/1,000. IRDye® 800CW-conjugated donkey anti-rabbit IgG (H+L) (LI-COR Biosciences) was used to detect primary antibodies using an Odyssey scanner (LI-COR Biosciences). Western blot zip files were uploaded onto Image Studio Lite Software (Version 5.2). Boxes were drawn around the bands with background boarder width set at 2 value for above and below the box. Intensity values minus background of pSTAT3 were divided by the values obtained for total STAT3, normalized to the average value obtained in control mice (SHAM+PBS) at the defined time-point, plotted, and significance calculated.

### Mouse Muscle Monocyte Isolation

All leukocytes were isolated from either SHAM-operated or SCI mice with an intramuscular injection of CDTX as described above. Injected hamstrings were harvested at 4 days post-surgery and muscle monocyte populations were isolated using a skeletal muscle dissociation kit (Miltenyi Biotech). In brief, hamstrings were cut into 1 mm pieces and up to 0.5 g of tissue was used per dissociation sample as per manufactures instructions. Total muscle leukocytes were subsequently separated into multiple populations using a Beckman Coulter Life Sciences CytoFLEX benchtop flow cytometer using the following antibodies (Biolegend); PerCP/Cyanine (CY) 5.5 anti-mouse/human CD11b (clone M1/70), FITC anti-mouse CD48 (clone HM48-1), APC anti-mouse F4/80 (clone BM8), Pacific Blue™ anti-mouse Ly-6C (clone HK1.4), APC/Cy7 anti-mouse Ly-6G (clone 1A8), and Zombie Aqua™ Fixable Viability Kit. Subsequently total muscle leukocytes were also sorted into multiple populations using a BD FACS Aria Fusion using the following antibodies (Biolegend): Brilliant Violet 785™ anti-mouse CD45 (clone 30-F11), FITC anti-mouse TER-119/Erythroid Cells (clone TER-119), FITC anti-mouse/human CD45R/B220 (clone RA3-6B2), FITC anti-mouse CD3ε (clone 145-2C11), APC anti-mouse F4/80 (clone BM8), Brilliant Violet 510™ anti-mouse/human CD11b (clone M1/70), PE anti-mouse Ly-6G (clone 1A8), APC/Cy7 anti-mouse CD48 (clone HM48-1), Pacific Blue™ anti-mouse Ly-6C (clone HK1.4), and 7-aminoactinomycin D (Life Technologies). Files were subsequently analyzed with Flow Jo software version 10.4. Muscle monocyte populations were sorted directly into trizol LS (ThermoFisher) and frozen until extraction.

### mRNA Extraction and qRT-PCR Analysis

For RNA isolation, frozen muscle was homogenized using a TissueRuptor (Qiagen), directly in Trizol (Life Technologies). After chloroform separation, RNA was isolated from aqueous phase. mRNA of sorted and cultured cells was isolated using chloroform separation followed by GeneJET RNA cleanup and concentration micro kit (ThermoFisher). Reverse transcription was performed using the iScript cDNA kit (BioRad) as per manufacturer's instructions. Analysis of mRNA expression for *Osm, Osmr, and Hprt* and was carried out using the Taqman Fast Advanced Master Mix and primer / probe sets (ThermoFisher): *Osmr* (Mm01307326_m1), *Osm* (Mm01193966_m1), and *Hprt* (Mm03024075_m1) on ViiA 7 Real-Time PCR System (Life Technologies) with PCR setting: 20 s at 95°C, then 40 cycles of 95°C (1 s) and 60°C (20 s). Results were normalized relative to *Hprt* mRNA expression.

### Micro-Computerized Tomography (μCT) and NHO Volume Quantification

NHO volume was measured *in vivo* or *ex vivo* using the Inveon positron emission tomography/computed tomography (PET-CT) multimodality system (Siemens Medical Solutions Inc.) as previously described (15). In brief, parameters were as follows: 360° rotation, 180 projections, 500 ms exposure time, 80 kV voltage, 500 μA current, and effective pixel size 36 μm. 3D reconstitutions were performed with the Inveon Research Workplace (Siemens Medical Solutions). To calculate NHO volumes, the region of interest (ROI) was drawn around the muscles containing NHO, and was carefully checked from three dimensions. After defining the ROI, the NHO region was defined by setting the threshold Hounsfield units (HU) to 450 HU.

### Histology

Fixed hind limbs were decalcified and processed as previously described (15). Five μm sections were cut and stained using Masson's Trichrome. In brief sections were deparaffinized and rehydrated then stained for 10 min in Weigert's iron hematoxylin, rinsed under tap water for 10 min, differentiated in 1% acid alcohol (1% hydrochloric acid) for 15–30 s, rinsed in tap water (3 min) then distilled water, followed by staining in Biebrich scarlet-acid fuchsine solution for 10–15 min (Biebrich scarlet, 1% aqueous, Acid fuchsine, 1% aqueous, glacial acetic acid), slides are then washed in distilled water and differentiated in 5% phosphomolybdic −5% phosphotungstic acid solution for 10–15 min or until collagen is not red. Slides were transferred into aniline blue solution for 5–10 min and rinsed in 1% acetic acid for 2–5 min, dehydrated, and mounted in resinous mounting medium. Immunohistochemistry was performed as previously described (15). Primary antibodies used were: rat anti-mouse F4/80 mAb (clone CI:A3-1, Abcam), rabbit anti-mouse Osterix/Sp7 polyclonal IgG (ab22552, Abcam), rabbit anti-mouse collagen type 1 polyclonal IgG (C7510-13, US Biological), or relevant isotype control antibodies; ratIgG2b (400602, Biolegend), or rabbit IgG (ab27478, Abcam). A 3-step procedure was employed using biotinylated F(ab)2 secondary antibodies (biotinylated goat anti-rat IgG and goat anti-rabbit IgG antibodies, Vector Labs) and VECTASTAIN Elite ABC -Peroxidase Kit (Vector Labs), was used to detect primary antibodies. Slides were viewed using an Olympus BX50 microscope with an attached DP26 camera and imaged using Olympus CellSens standard 1.7 imaging software (Olympus).

### Quantification of F4/80 Immunohistochemistry

Immunohistochemistry staining for the pan macrophage marker F4/80 was imaged using an Olympus VS120 (Olympus) at 40X magnification. Automated digital image analysis was subsequently performed using the Visiopharm Integrator System (Visiopharm, Hoersholm, Denmark). In each sample, at each sectional depth (4 depths analyzed with each depth at least 50 μm apart), ROIs were generated which contained all damaged muscle. Automated analysis was performed from the ROIs and the data was calculated as percent of F4/80^+^ staining per total area of injured muscle. All cases were visually reviewed to ensure accuracy. Data was represented separately at each sectional depth as the area of damaged tissue is not uniform throughout each hamstring.

### Statistical Analysis

Statistically significant differences were determined using ANOVA with *post-hoc* Tukey's multiple comparison test or Mann-Whitney test using PRISM 6 or 7 (GraphPad software, La Jolla, CA, USA).

## Results

### Increased Ly6C^high^ Monocyte Infiltration in Injured Muscles of Mice Following SCI

**Figure 1:**
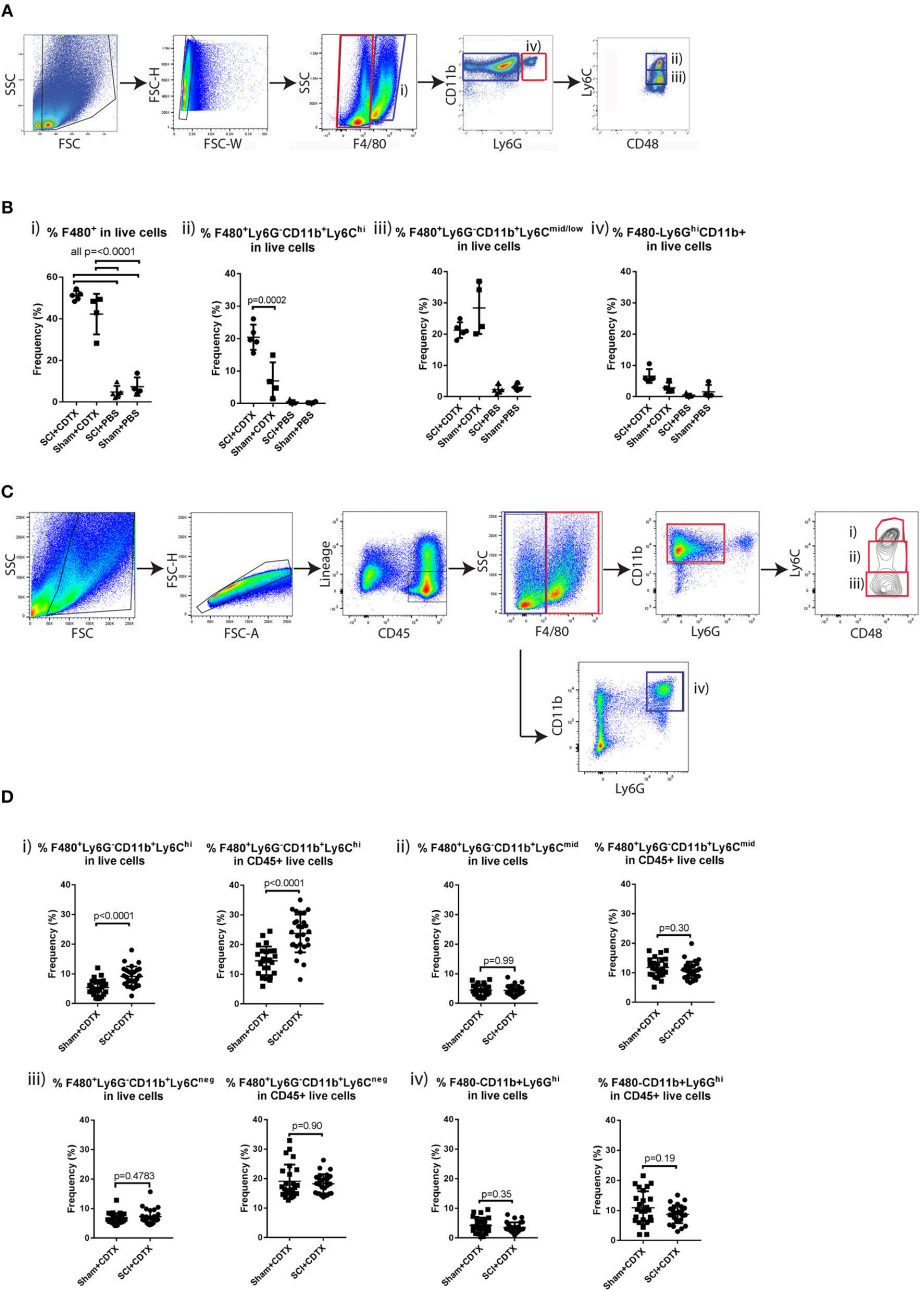
Increased inflammatory monocyte infiltration in injured muscles of mice developing NHO. **(A)** C57BL/6 mice underwent either SCI or Sham surgery followed by an intra muscular injection of CDTX or PBS. Muscle leukocytes were extracted from hamstrings at day 4 post-surgery and isolated into four subsets based on forward scatter, side scatter as well as zombie aqua negativity (viable cells), F4/80, Ly6G, CD11b, and Ly6C expression. **(B)** Frequencies of each leukocyte population relative to total live muscle cells using the gating strategy outlined: (i) “Total F4/80^+^ cells” (CD11b^+^F4/80^+^, blue gates in **A**), (ii) “Ly6C^hi^ inflammatory monocytes” (F4/80^+^CD11b^+^Ly6G^−^ CD48^+^Ly6C^hi^, blue gates in **A**), (iii) “Ly6C^mid/lo^ monocytes/macrophages” (F4/80^+^ CD11b^+^ Ly6G^−^CD48^+^ Ly6C^mid/lo^, blue gates in **A**), and (iv) “granulocytes” (F4/80^−^CD11b^+^Ly6G^+^, red gates in **A**). The frequency of Ly6C^hi^ inflammatory monocytes (relative to total live muscle cells) in mice developing NHO (SCI+CDTX), compared to all other treatment groups was significantly higher (*p* = 0.0002 ANOVA *n* = 3–5/group). Each dot represents an individual mouse. Bars represent as mean ± SD. **(C)** Muscle leukocytes were extracted from hamstrings at day 4 post surgery, and 4 separate leukocyte populations were identified by flow cytometry using the following gating strategy: (i) “Ly6C^hi^ inflammatory monocytes” as CD45^+^ lineage (Ter119,CD3ε,B220)-negative F4/80^+^ CD11b^+^ Ly6G^−^ CD48^+^Ly6C^hi^, red gates, (ii) “Ly6C^mid^ monocytes/macrophages” CD45^+^ Lin^−^ F4/80^+^ CD11b^+^ Ly6G^−^CD48^+^ Ly6C^mid^, red gates, (iii) “Ly6C^neg^ monocytes” CD45^+^ Lin^−^ F4/80^+^ CD11b^+^ Ly6G^−^ CD48^+^ Ly6C^neg^ red gates, and (iv) “granulocytes” CD45^+^ Lin^−^ F4/80^neg^ CD11b^+^ Ly6G^+^, blue gates. **(D)** Frequencies of each myeloid subset relative to total live muscle cells and to total live CD45^+^ muscle leukocytes. There was a significant increase in frequency of Ly6C^hi^ monocytes relative to total live muscle cells (Di, *p* < 0.0001 Mann-Whitney test) and to CD45^+^ live muscle leukocytes (Di, *p* < 0.0001 Mann-Whitney test) after SCI+CDTX compared to Sham+CDTX. Each dot represents an individual mouse, *n* = 25–27/treatment group. Bars represent mean ± SD.

We have previously reported that systemic depletion of phagocytic macrophages and monocytes by injections of clodronate-loaded liposomes significantly reduces NHO development (13). Therefore, we further characterized the macrophage/monocyte populations present within the muscles of mice developing NHO by flow cytometry. In this model, only mice that undergo both SCI + i.m. CDTX injection develop NHO exclusively in the CDTX injected muscle (13, 15). Mice underwent SCI or SHAM surgery followed by an intramuscular injection of CDTX to cause muscle injury or a control PBS injection. At 4 days post-surgery leukocytes were extracted from the hamstrings of all mice and isolated into four subsets based on forward scatter, side scatter as well as zombie aqua negativity (viable cells), F4/80, Ly6G, CD11b, and Ly6C expression (Figure 1A). Preliminary flow cytometry analysis confirmed that CDTX-induced intramuscular injury caused a large and significant accumulation of total F4/80^+^ monocyte/macrophages (Figure 1Bi *p* < 0.0001) in SCI+CDTX and SHAM+CDTX groups compared to control groups (SCI+PBS and SHAM+PBS). When the total F4/80^+^ population was sub-gated based on expression of Ly6C, we observed a significantly higher frequency of Ly6C^high^ ‘inflammatory monocytes/macrophages’ (CD11b^+^F4/80^+^Ly6G^−^Ly6C^hi^) in the mice that develop NHO (SCI+CDTX), compared to SHAM+CDTX mice (Figure 1Bii *p* = 0.0002). Minimal Ly6C^hi^ monocyte/macrophages were noted in the SCI+PBS and SHAM+PBS groups. The frequencies of Ly6C^mid/lo^ monocyte/macrophages (CD11b^+^F4/80^+^Ly6G^−^Ly6C^mid^/^lo^) was unchanged between SCI+CDTX and SHAM+CDTX groups (Figure 1Biii). We also observed the presence of granulocytes (CD11b^+^F4/80^−^Ly6G^hi^) (Figure 1Biv**)**, albeit with lower frequencies compared to monocyte/macrophage subsets. In view of these preliminary results, we focused our subsequent analysis on leukocyte populations infiltrating the CDTX-injured muscles in SCI+CDTX and SHAM+CDTX groups in a larger cohort of mice to achieve higher statistical power. Leukocytes were isolated into 4 separate populations based on forward scatter, side scatter as well as 7-actinomycin D- negativity (7AAD^−^ viable cells), CD45, lineage (Ter119, CD3ε, B220), F4/80, Ly6G, CD11b, Ly6C expression (Figure 1C). Although we initially wanted to incorporate antibodies specific for CD169, Mer tyrosine kinase and VCAM-1 which clearly identifies macrophages from monocytes (31), preliminary experiments on bone marrow cells where macrophages and monocytes are abundant confirmed this was not possible as these antigens are cleaved from the cell surface by the protease cocktail used to extract leukocytes from skeletal muscles (data not shown). Despite this limitation, we again observed that the frequency of “Ly6C^hi^ inflammatory monocytes/macrophages” (CD45^+^Lin^−^CD11b^+^F4/80^+^Ly6G^−^Ly6C^hi^) in live muscle cells was significantly higher in mice that develop NHO (SCI+CDTX) mice compared to SHAM+CDTX mice (Figure 1Di, left panel *p* < 0.0001). When the frequency of Ly6C^hi^ monocytes was calculated relative to all CD45^+^ leukocytes present, the frequency of these inflammatory monocytes was also significantly increased (Figure 1Di, right panel *p* < 0.0001). The frequencies of other monocyte/macrophage subsets identified as CD45^+^Lin^−^CD11b^+^F4/80^+^Ly6G^−^Ly6C^mid^ monocytes (Figure 1Dii), CD45^+^Lin^−^CD11b^+^F4/80^+^Ly6G^−^Ly6C^neg^ monocytes (Figure 1Diii), as well as CD45^+^Lin^−^CD11b^+^F4/80^−^Ly6G^+^ neutrophils (Figure 1Div) were not significantly different in SCI+CDTX compared to SHAM+CDTX mice.

### Expression of OSM and OSMR in Muscle Cell Populations

**Figure 2:**
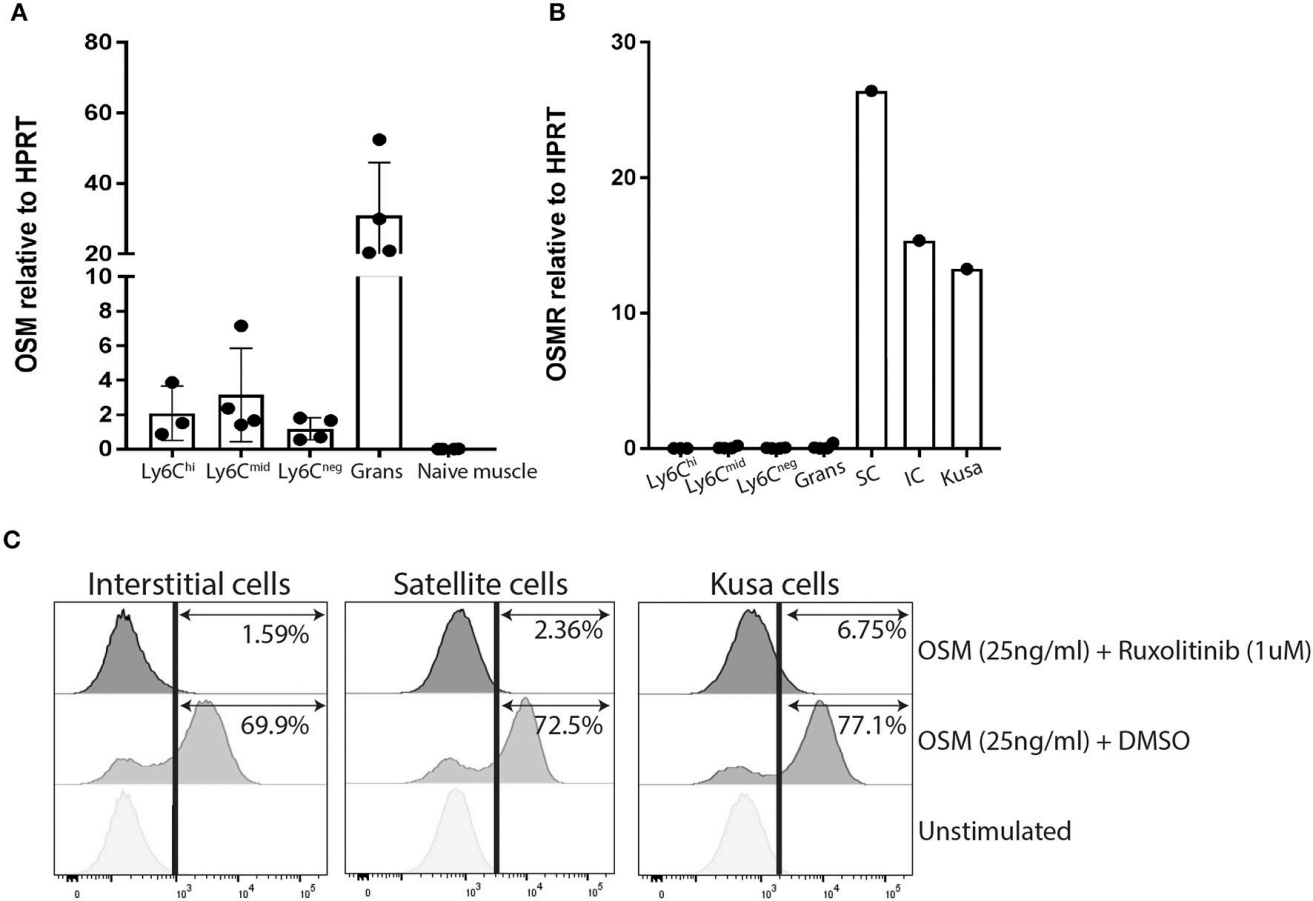
OSM and OSMR expression and signaling in muscle cells. **(A)** *Osm* mRNA expression by qRT-PCR. *Osm* is expressed by all sorted infiltrating myeloid populations but absent in whole naïve skeletal muscle. Each dot represents an individual mouse (*n* = 3–4/group). Bars represent mean ± SD. **(B)** *Osmr* mRNA expression is only present in sorted mouse muscle satellite and interstitial cells, not in any sorted myeloid population infiltrating the injured muscle **(C)** Phos-flow of pSTAT3 Y705 phosphorylation in cultured CD45^−^Ter119^−^CD31^−^CD34^+^Sca1^+^ muscle interstitial cells (IC), CD45^−^Ter119^−^CD31^−^CD34^+^Sca1^−^ muscle satellite cells (SC), and Kusa4b10 cells. Cells were incubated for 10 min at 37°C with medium alone (unstimulated), or 25 ng/ml recombinant mouse OSM plus DMSO or of 25 ng/ml OSM plus 1 μM ruxolitinib. Cell were then fixed and permeabilized before staining with AlexaFluor647-conjugated mouse mAb anti-pSTAT3 (Y705) and analyzed by flow cytometry. Representative figures were one of two independent experiments.

We have previously confirmed by qRT-PCR, that *Osm* mRNA is significantly upregulated in the whole muscles of mice developing NHO (15). As there is no commercial monoclonal antibody specific for mouse OSMR that works by flow cytometry, we isolated RNA from the sorted myeloid populations described in Figure 1D at 4 days post-surgery, as well as whole skeletal muscle from naïve mice and mouse muscle progenitor cell populations; CD45^−^Ter119^−^CD31^−^CD34^+^Sca1^−^ satellite cells and CD45^−^Ter119^−^CD31^−^CD34^+^Sca1^+^ interstitial cells [also known as fibro-adipogenic progenitors or FAP (32)] freshly sorted from naïve skeletal muscle, to establish which cell types express *Osm* and *Osmr* mRNA. *Osm* mRNA was detected in all myeloid populations infiltrating the SCI+CDTX-injured muscle with the highest abundance in granulocytes (Figure 2A), whereas no *Osm* was detected in whole naïve skeletal muscle. These results are consistent with the tissue expression profile of mouse *Osm* mRNA in the BioGPS database (http://biogps.org). In sharp contrast, *Osmr* mRNA was undetectable in myeloid populations in the muscle but was expressed by both muscle satellite cells and interstitial cells (Figure 2B). This suggests that OSM can act directly on muscle progenitor cells that express its receptor OSMR rather than indirectly via infiltrating myeloid cells.

### OSM Induces STAT3 Y705 Phosphorylation in Cultured Mouse Muscle Cells

Given that both muscle satellite cells and interstitial cells expressed *Osmr* mRNA, we further investigated OSM/OSMR signaling in these cells. The OSMR/gp130 receptor complex is known to activate both JAK1 and JAK2 tyrosine kinases following OSM binding (23, 24). Once activated, JAK1 and JAK2 tyrosine phosphorylate STAT1 and STAT3 (23) which enable their nuclear translocation to initiate transcription of OSM responsive genes. In preliminary experiments, we were unable to detect phosphorylated JAK1 and JAK2 by immunoprecipitation and western-blot of whole muscle lysates because the very low levels of total JAK1 and JAK2 proteins (data not shown). Instead, we measured the tyrosine phosphorylation status of the JAK1/2 substrate STAT3 in response to recombinant mouse OSM in satellite and interstitial cells sorted from the muscles of naïve mice. The mouse mesenchymal progenitor cell line Kusa4b10 (29, 30) was used as a positive control. By flow cytometry with a mAb specific for STAT3 phosphorylation on tyrosine 705 (pSTAT3 Y705), we confirmed that recombinant mouse OSM caused a rapid phosphorylation of STAT3 Y705 on all cell types tested (Figure 2C), confirming that OSMR is functional on mouse muscle satellite and interstitial cells. To confirm that STAT3 Y705 phosphorylation was mediated by JAK1/2, we also preincubated cells for 30 min with the small synthetic JAK1/2 inhibitor ruxolitinib (27, 28). Ruxolitinib completely inhibited phosphorylation of STAT3 Y705 in response to mouse OSM in all three cell types (Figure 2C). Together these results suggest that OSMR expressed by satellite and interstitial cells are able to respond to upregulated OSM in muscle and activate down-stream JAK1/2-STAT3 signaling pathway.

### Persistence of STAT3 Tyrosine Phosphorylation in the Injured Muscle Following SCI

**Figure 3:**
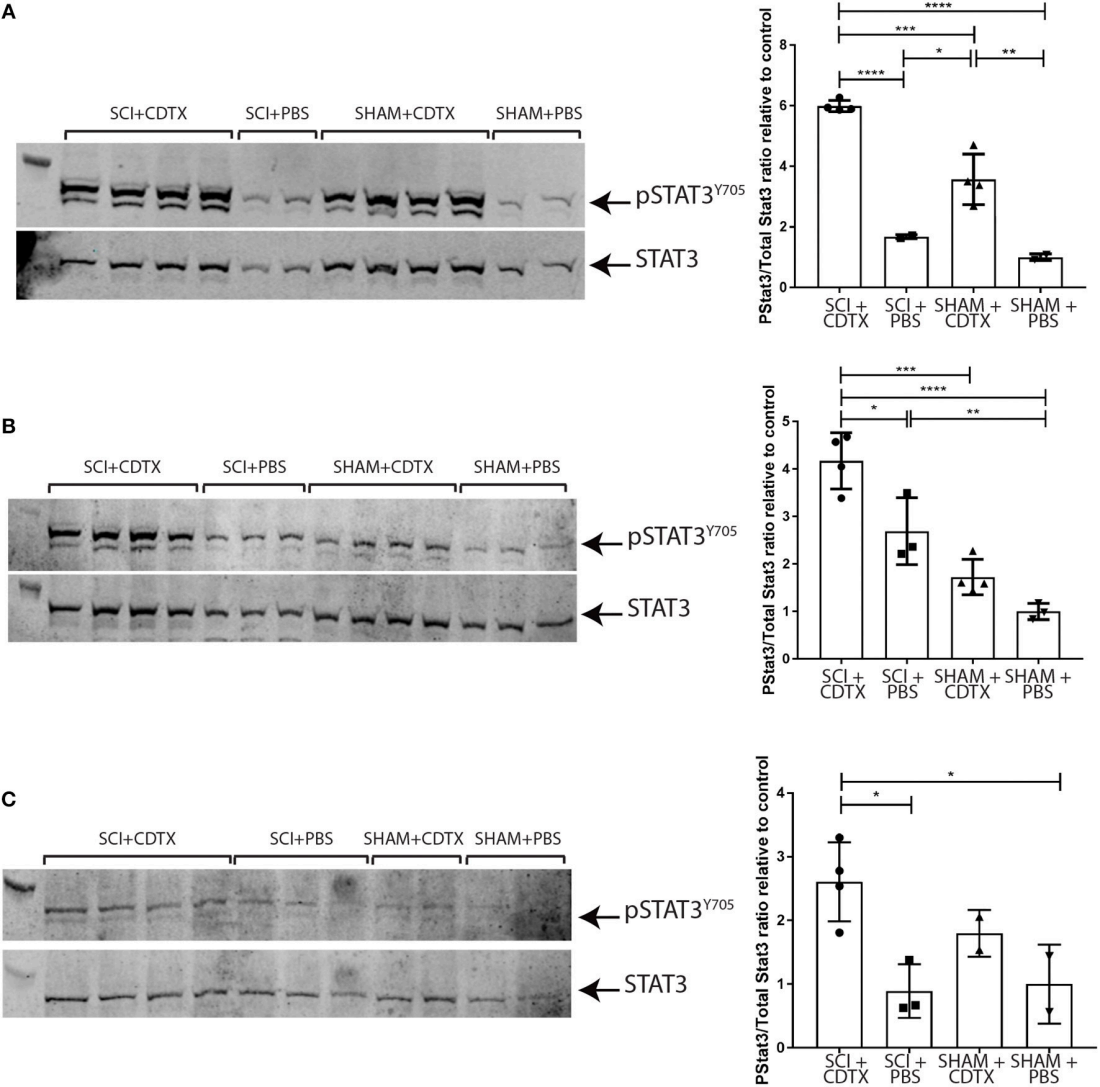
Persistence of STAT3 tyrosine phosphorylation in injured muscles following spinal cord injury. Western-blots of whole muscle lysates collected from mice that underwent either SCI or SHAM surgery together with an intramuscular injection of either CDTX or PBS. Western blot of whole muscle lysates from individual mice taken on day 4 **(A)**, 7 **(B)**, or 14 **(C)** post-injury. Each membrane was probed with rabbit anti-pSTAT3 Y705 mAb, stripped and then re-probed with rabbit anti-total STAT3 mAb. Band fluorescence intensity was quantified and ratio of signal intensity of pSTAT3 vs. total STAT3 calculated for each individual mouse and normalized relative to the average pSTAT3/STAT3 ratio in control mice (SHAM+PBS) at each time-point. Each lane and each dot represents a separate mouse, *n* = 2–4 mice/treatment/time-point. Bars represent mean ± SD. *P* values were calculated by ANOVA, ^*^p < 0.05, ^**^p < 0.01, ^***^p < 0.001, ^****^p < 0.0001.

Next, we examined STAT3 Y705 phosphorylation in the muscles of mice developing NHO. Western blots for pSTAT3 Y705 and total STAT3 were performed using whole muscle lysates from hamstrings of mice that underwent either (1) SCI+CDTX, (2) SCI+PBS, (3) SHAM+CDTX, or (4) SHAM+PBS at day 4, 7, and 14 days post-surgery. At 4 days post-surgery there was a clear increase in STAT3 Y705 phosphorylation in muscle injured with CDTX (Figure 3A). Importantly the ratio of pSTAT3 Y705 vs. total STAT3 normalized to the pSTAT3/STAT3 ratio measured in control mice (SHAM+PBS), was significantly higher in SCI+CDTX mice compared to SHAM+CDTX mice (Figure 3A). Seven days post-injury, STAT3 Y705 phosphorylation persisted in the injured muscles from the SCI+CDTX group while it was resolving in the SHAM+CDTX group (Figure 3B). This pattern of persistent STAT3 Y705 phosphorylation in the injured muscles of the SCI+CDTX group that developed NHO was noted up to 14 days post-surgery (Figure 3C) whereas in SHAM+CDTX, pSTAT3 Y705 had returned to levels not significantly different from those observed in controls without muscle injury as expected from previous reports showing that without a SCI, CDTX-injured muscles are mostly repaired 14 post-injury (33). These data establish that the combination of SCI with muscle injury, compared to all other control treatments, caused enhanced STAT3 signaling in the injured muscle which persisted for an extended period of time after the initial injury. Overall these data establish that in the context of a SCI, STAT3 phosphorylation is exaggerated and persists over a longer period of time in injured muscles developing NHO.

### JAK1/2 Inhibition Significantly Reduced NHO Development Following SCI

**Figure 4:**
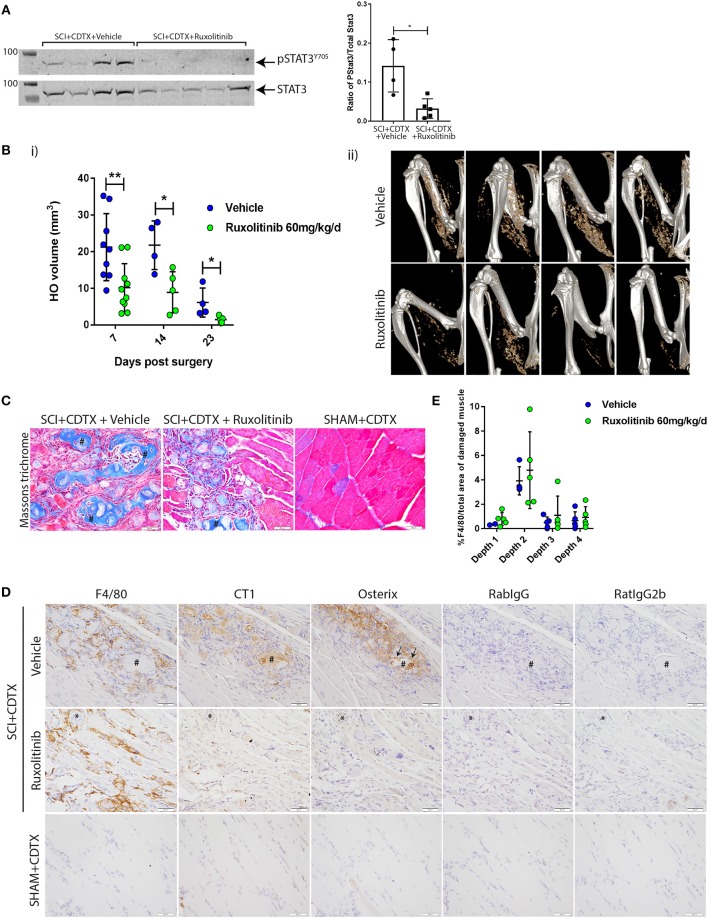
Inhibition of JAK1/2 kinases with ruxolitinib reduces NHO development after SCI *in vivo*. **(A)** Western-blots of whole muscle lysates collected at day 7 from SCI+CDTX mice that were treated with either vehicle control or ruxolitinib (60 mg/kg bi-daily) from day 0–7 post surgery. Western-blots of whole muscle lysates were probed with rabbit anti-pSTAT3 Y705 mAb, and rabbit anti-total STAT3 mAb, then band fluorescence was quantified and ratio of signal intensity of pSTAT3 versus total STAT3 calculated for each individual mouse. Each lane and each dot represents a separate mouse, *n* = 4–5/treatment group. Data represented as mean ± SD, ^*^p = 0.03 by Mann-Whitney test. **(B)** Measurement of NHO volume by micro CT (μCT) in mice which received SCI+CDTX and treated with either vehicle control or ruxolitinib (60 mg/kg bi-daily) from day 0–7 post surgery. (i) NHO volumes were quantified *in vivo* by μCT at indicated time points post-surgery illustrating the reduction in NHO development. Each dot represents a separate mouse, *n* = 4–10 mice/treatment/time point. Data represented as mean ± SD, ^**^p = 0.0076, ^*^p = 0.031, and *p* = 0.015 respectively by Mann-Whitney test. (ii) Representative μCT images at 7 days post-surgery **(C)** Masson's trichrome staining 3 weeks post-surgery confirming the development of multiple NHO bone and collagen+ foci (crosshatches) within the muscle in vehicle treated mice, which are reduced after ruxolitinib treatment, and absent in control mice (SHAM+CDTX) **(D)** Immunohistochemistry staining of serial sections from SCI+CDTX mice 3 weeks post-surgery (top and middle panels). Mice were treated with vehicle or ruxolitinib (60 mg/kg bi-daily) from day 0–7 post surgery. Stains were performed with either rat anti-F4/80 mAb, rabbit anti-collagen I (CT1), or anti-osterix antibodies. Isotype control (rat IgG2b for F4/80; Rabbit IgG for CT1, and Osterix) are also shown to confirm specificity of staining. In vehicle treated mice CT1^+^ NHO foci are present within the damaged muscle (crosshatch), these foci are surrounded by F4/80^+^ macrophages and have Osterix^+^ cells lining the NHO foci surface (arrows). After ruxolitinib treatment there are still F4/80^+^ macrophages within the damaged muscle, however there are less CT1^+^ NHO foci with Osterix^+^ cells lining the surface. ^*^symbols denote the same anatomical landmark in each image. NHO development is absent in SHAM+CDTX mice 3 weeks post-surgery, with no CT1, and Osterix expression (bottom panel). **(E)** Quantification of F4/80 expression via IHC confirmed that ruxolitinib treatment did not change F4/80^+^ macrophage expression within the hamstrings of vehicle vs. ruxolitinib treated mice, 7 days post-surgery. Each dot represents a separate mouse, *n* = 2–5/treatment group/sectional depth. Four different depths were analyzed for each sample with at least 50 μm between each depth. Data represented as mean ± SD. All images taken at 40X magnification, scale bar represents 50 μm.

From this we hypothesized that exaggerated and persistent STAT3 tyrosine phosphorylation and activation by JAK1/2 in injured muscles of SCI mice is functionally important in NHO pathogenesis. To test this, we treated mice that underwent SCI+CDTX with ruxolitinib bi-daily for the first 7 days post-surgery. Ruxolitinib treatment significantly reduced STAT3 Y705 phosphorylation at 7 days post-surgery (Figure 4A ^*^p = 0.03). Micro CT (μCT) confirmed a significant reduction in NHO bone volume at day 7, 14, and 23 post surgery in mice treated with ruxolitinib for the first 7 days post-injury (Figure 4Bi,ii ^**^p = 0.0076, ^*^p = 0.031, and ^*^p = 0.015). Visual representation of collagen^+^ NHO by Masson's trichrome staining at 3 weeks post-surgery was consistent with μCT quantification and confirmed the presence of collagen^+^ NHO foci in the muscles of vehicle treated SCI+CDTX mice (Figure 4C, crosshatches). Collagen^+^ NHO foci were reduced after ruxolitinib treatment. As previously described by us (13, 15) and others (33), in SHAM+CDTX mice at 3 weeks post-surgery, no NHO and little collagen deposition was observed, with reformation of muscle fibers confirming that in the absence of SCI, the CDTX-injured muscle repairs within 7–14 days post-injury (33). Immunohistochemistry with anti-collagen type I or anti-osterix/SP7 antibodies were consistent with both μCT and Masson's trichrome staining and confirmed that in vehicle treated SCI+CDTX mice the collagen type I^+^ NHO foci were lined with osterix^+^ osteo-lineage cells (Figure 4D, top panel, arrows). Reduced osterix-positive osteo-lineage cells and type 1 collagen deposition was noted after ruxolitinib treatment (Figure 4D, middle panel). In SHAM+CDTX mice there was little expression of collagen type I and osterix (Figure 4D, bottom panel). Ruxolitinib treatment had minimal impact on the density of F4/80^+^ macrophages within the injured muscles 7 days post-surgery (Figure 4E).

## Discussion

The inflammatory component that frequently accompanies severe trauma of the central nervous system and the spine has been suggested to be a key factor in NHO development (5, 34, 35). We have previously established in a mouse model of SCI-induced NHO in which macrophages infiltrate the damaged muscles was critical for NHO development (13–15). Our current study demonstrates that SCI causes an increased infiltration of Ly6C^hi^ inflammatory monocytes/macrophages into injured muscles. Furthermore, we show that myeloid cells infiltrating the injured muscle express the pro-inflammatory cytokine OSM. Binding of OSM to OSMR, which is expressed by satellite cells and interstitial cells isolated from muscle, activates JAK1/2 tyrosine kinases with subsequent STAT3 tyrosine phosphorylation and activation *in vitro*. *In vivo* we established that SCI with accompanying muscle injury caused an increase in STAT3 phosphorylation in injured muscles which persisted for up to 2 weeks only in the muscles that develop NHO. Finally, *in vivo* inhibition of JAK1/2 with ruxolitinib reduced STAT3 phosphorylation in injured muscles and most importantly reduced NHO volume subsequent to SCI combined with muscle injury.

OSM has been recently reported to induce muscle satellite cell quiescence *in vivo*, and conditional deletion of the *Osmr* gene specifically in satellite cells led to reduced myofiber regeneration in response to injury (36). In agreement with this, STAT3, which is activated by JAK1/2 immediately downstream of the OSMR:gp130 complex, has been implicated in controlling satellite cell expansion and muscle repair after muscle injury (37). In addition, STAT3 knock-down or conditional *Stat3* gene deletion in satellite cells increased satellite cell proliferation following muscle injury but impaired muscle repair, suggesting that STAT3 activation is required for accelerated muscle repair (37, 38). In contrast, OSM is known to promote osteogenic differentiation of mesenchymal stromal cells *in vitro* (18, 39) and osteoblast differentiation and bone formation *in vivo* (16, 17, 19). Therefore, OSM plays an important role in both muscle repair and osteogenic differentiation and activity. This is consistent with our observations that (1) OSMR is expressed in skeletal muscles by both satellite cells, which regenerate myoblasts following muscle injury (40), and by interstitial cells which are of mesenchymal origin (32), and (2) that OSM causes STAT3 phosphorylation in both cell types *in vitro*. A limitation of our study is that we were unable to demonstrate STAT3 phosphorylation specifically in muscle satellite cells or interstitial cells *in vivo*. The extended enzymatic digestion of muscles at 37°C required to obtain single cell suspension amenable for flow cytometry, removes OSM protein from the extracellular milieu thus disrupting OSMR ligation on satellite and interstitial cells and downstream JAK/STAT signaling. Further immunohistological experiments using reporter mice in which muscle satellite cells or mesenchymal cells are specifically labeled with a fluorescent reporter will be required to definitively prove STAT3 activation *in vivo* in these two cell types.

It is also important to note that in our experiments, ruxolitinib may also block STAT activation in additional cell types particularly myeloid cells that infiltrate the injured muscle. Indeed, these myeloid cells express receptors for other inflammatory cytokines such as IL-6 (41), granulocyte colony-stimulating factor (G-CSF) (42) and granulocyte macrophage colony-stimulating factors (GM-CSF) (43) which also activate STATs via JAK1/2. Although it remains to be determined whether IL-6, G-CSF, and GM-CSF contribute to NHO pathogenesis (44), the ruxolitinib-mediated inhibition of JAK1/2 in both muscle progenitor cells and infiltrating myeloid cells may also contribute to the overall inhibition of NHO in our model.

Intriguingly, while STAT3 inhibition has been reported to improve muscle repair in mice without SCI (37, 38), we did not note improved muscle repair following JAK1/2 inhibitor administration in mice that underwent SCI as macrophage infiltration and collagen deposits remained in the injured muscles even after ruxolitinib treatment. A few hypotheses can be formulated to explain this divergent outcome in terms of muscle repair. Firstly, the SCI may cause a dramatic change in the function of macrophages orchestrating muscle repair (33) and in muscle satellite and interstitial cells, such that inhibition of either STAT3 or JAK1/2 is not sufficient to re-establish coordinated muscle regeneration. A second possibility is that the JAK1/2 inhibitor used in our study also inhibits the activation of other STAT proteins such as STAT1 and STAT5 which may be activated in response to OSM (16) as well as STAT activation in response to other cytokines such IL-6, IL-12, G-CSF, or GM-CSF in muscle cells and macrophages which orchestrate muscle regeneration as discussed above.

It is also important to note that despite the significant effect of ruxolinitib in reducing STAT3 phosphorylation in muscles of mice developing NHO, and the reduction in NHO volume after ruxolitinib treatment, while pronounced was only partial (Figure 4). Herein we find that SCI and muscle injury caused an exaggerated infiltration of Ly6C^hi^ inflammatory monocytes into the injured muscles consistent with our previous observation that *in vivo* depletion of phagocytes with clodronate-loaded liposomes markedly inhibited NHO formation (13). Because of this increased inflammatory monocyte infiltration, it is likely that other pro-inflammatory cytokines and mediators are released within the injured muscle and participate to NHO development. For instance we have previously reported in this model of SCI-induced NHO that substance P may contribute to NHO development (13). Others have reported in a rat model of multi-trauma, that the retinoic acid receptor-γ agonist Palovarotene also partially decreased heterotopic ossification (45). In a similar rat model of multi-trauma, rapamycin was also found to partially decrease heterotopic ossification, suggesting that mammalian targets of rapamycin (mTOR) may also play an important role (46). Altogether these findings suggest that many other pathways could be abnormally activated in injured muscles in the context of a SCI. Indeed since NHO is driven by two different insults, it is unlikely that the pathogenesis converges to a single pathway. Therefore, a highly effective therapy is likely to require a combined approach. We are currently undertaking transcriptome analyses on injured muscles with and without SCI to elucidate the molecular pathways that could potentially promote NHO development.

Although ruxolitinib caused a pronounced but still partial reduction in NHO development, inhibitors of JAK1/2 or STAT3 may represent a new therapeutic approach to decrease NHO development in patients or perhaps to avoid NHO re-occurrence after surgical resection, which is observed in 6% of NHO patients (2, 9, 12). However, the roles of STAT3 and JAK1/2 in spinal cord recovery will need to be carefully evaluated. In a rat model of SCI, treatment with a STAT3 inhibitor post-surgery promoted neural stem cell differentiation (47). In other studies, augmentation of IL-6 after SCI resulted in enhanced infiltration of neutrophils and macrophages with a subsequent increase in lesion size and reduction in axonal growth, suggesting that balanced IL-6 signaling is required for efficient repair after SCI (48). However, other studies have demonstrated that conditional deletion of STAT3 in reactive astrocytes leads to the limited migration of astrocytes, higher infiltration of inflammatory cells, demyelination, and more severe loss of motor function following SCI (49, 50). The SCI-NHO model used in our studies involves a complete spinal cord transection and further analysis on neurological recovery was not possible in this model. Therefore, further studies in a SCI-NHO model where neurological recovery is achievable are necessary to effectively determine whether ruxolitinib treatment to reduce NHO development has any beneficial or negative impact on neurological recovery following SCI.

In conclusion our experiments suggest that STAT3 activation persists in the muscles of mice that are developing NHO following SCI and that targeting STAT3 activation via transient JAK1/2 inhibition immediately following SCI may be a possible therapeutic approach to reduce NHO development in patients.

## Data Availability

The datasets generated for this study are available on request to the corresponding author.

## Ethics Statement

This study was carried out in accordance with the Australian Code of Animal Experimentations. All protocols and experiments were approved by the Animal Experimentation Committee of the University of Queensland, Saint Lucia, Queensland, Australia.

## Author Contributions

KA, H-WT, WF, BJ, MS, IK, and SM performed the experiments. KA, H-WT, and J-PL conceived the experiments and the work. KA, H-WT, and J-PL wrote the manuscript. KA, H-WT, FG, ARP, and J-PL edited the manuscript.

### Conflict of Interest Statement

The authors declare that the research was conducted in the absence of any commercial or financial relationships that could be construed as a potential conflict of interest.

## References

[1] Ohlmeier M Suero EM Aach M Meindl R Schildhauer TA Citak M. Muscle localization of heterotopic ossification following spinal cord injury. Spine J. (2017) 17:1519–22. 10.1016/j.spinee.2017.04.021 28456672

[2] Genet F Jourdan C Schnitzler A Lautridou C Guillemot D Judet T. Troublesome heterotopic ossification after central nervous system damage: a survey of 570 surgeries. PLoS ONE. (2011) 6:e16632. 10.1371/journal.pone.0016632 21304993 PMC3031592

[3] Dizdar D Tiftik T Kara M Tunc H Ersoz M Akkus S. Risk factors for developing heterotopic ossification in patients with traumatic brain injury. Brain Inj. (2013) 27:807–11. 10.3109/02699052.2013.775490 23730889

[4] Reznik JE Biros E Marshall R Jelbart M Milanese S Gordon S. Prevalence and risk-factors of neurogenic heterotopic ossification in traumatic spinal cord and traumatic brain injured patients admitted to specialised units in Australia. J Musculoskelet Neuronal Interact. (2014) 14:19–28. 24583537

[5] Brady RD Shultz SR McDonald SJ O'Brien TJ. Neurological heterotopic ossification: current understanding and future directions. Bone. (2018) 109:35–42. 10.1016/j.bone.2017.05.015 28526267

[6] Forsberg JA Pepek JM Wagner S Wilson K Flint J Andersen RC. Heterotopic ossification in high-energy wartime extremity injuries: prevalence and risk factors. J Bone Joint Surg Am. (2009) 91:1084–91. 10.2106/JBJS.H.00792 19411456

[7] Vanden Bossche L Vanderstraeten G. Heterotopic ossification: a review. J Rehabil Med. (2005) 37:129–36. 10.1080/16501970510027628 16040468

[8] Salga M Jourdan C Durand MC Hangard C Denormandie P Carlier RY. Sciatic nerve compression by neurogenic heterotopic ossification: use of CT to determine surgical indications. Skeletal Radiol. (2015) 44:233–40. 10.1007/s00256-014-2003-6 25218150

[9] Genet F Chehensse C Jourdan C Lautridou C Denormandie P Schnitzler A. Impact of the operative delay and the degree of neurologic sequelae on recurrence of excised heterotopic ossification in patients with traumatic brain injury. J Head Trauma Rehabil. (2012) 27:443–8. 10.1097/HTR.0b013e31822b54ba 22495100

[10] Genet F Marmorat JL Lautridou C Schnitzler A Mailhan L Denormandie P. Impact of late surgical intervention on heterotopic ossification of the hip after traumatic neurological injury. J Bone Joint Surg Br. (2009) 91:1493–8. 10.1302/0301-620X.91B11.22305 19880896

[11] Genêt F Denormandie P Keenan MA. Orthopaedic surgery for patients with central nervous system lesions: concepts and techniques. Ann Phys Rehabil Med. (2018) 10.1016/j.rehab.2018.09.004 30290282

[12] Genet F Jourdan C Lautridou C Chehensse C Minooee K Denormandie P. The impact of preoperative hip heterotopic ossification extent on recurrence in patients with head and spinal cord injury: a case control study. PLoS ONE. (2011) 6:e23129. 10.1371/journal.pone.0023129 21853078 PMC3154269

[13] Genet F Kulina I Vaquette C Torossian F Millard S Pettit AR. Neurological heterotopic ossification following spinal cord injury is triggered by macrophage-mediated inflammation in muscle. J Pathol. (2015) 236:229–40. 10.1002/path.4519 25712044

[14] Levesque J-P Sims NA Pettit AR Alexander KA Tseng H-W Torossian F. Macrophages driving heterotopic ossification: convergence of genetically-driven and trauma-driven mechanisms. J Bone Miner Res. (2018) 33:365–6. 10.1002/jbmr.3346 29178621

[15] Torossian F Guerton B Anginot A Alexander KA Desterke C Soave S. Macrophage-derived oncostatin M contributes to human and mouse neurogenic heterotopic ossifications. JCI Insight. (2017) 2:e96034. 10.1172/jci.insight.96034 29093266 PMC5752299

[16] Walker EC McGregor NE Poulton IJ Solano M Pompolo S Fernandes TJ. Oncostatin M promotes bone formation independently of resorption when signaling through leukemia inhibitory factor receptor in mice. J Clin Invest. (2010) 120:582–92. 10.1172/JCI40568 20051625 PMC2810087

[17] Sims NA Quinn JM. Osteoimmunology: oncostatin M as a pleiotropic regulator of bone formation and resorption in health and disease. Bonekey Rep. (2014) 3:527. 10.1038/bonekey.2014.22 24876928 PMC4037876

[18] Guihard P Danger Y Brounais B David E Brion R Delecrin J. Induction of osteogenesis in mesenchymal stem cells by activated monocytes/macrophages depends on oncostatin M signaling. Stem Cells. (2012) 30:762–72. 10.1002/stem.1040 22267310

[19] Guihard P Boutet MA Brounais-Le Royer B Gamblin AL Amiaud J Renaud A. Oncostatin M, an inflammatory cytokine produced by macrophages, supports intramembranous bone healing in a mouse model of tibia injury. Am J Pathol. (2015) 185:765–75. 10.1016/j.ajpath.2014.11.008 25559270

[20] Gerhartz C Heesel B Sasse J Hemmann U Landgraf C Schneider-Mergener J. Differential activation of acute phase response factor/STAT3 and STAT1 via the cytoplasmic domain of the interleukin 6 signal transducer gp130: I. Definition of a novel phosphotyrosine motif mediating STAT1 activation. J Biol Chem. (1996) 271:12991–8. 10.1074/jbc.271.22.12991 8662591

[21] Heinrich PC Behrmann I Muller-Newen G Schaper F Graeve L. Interleukin-6-type cytokine signalling through the gp130/Jak/STAT pathway. Biochem J. (1998) 334:297–314. 10.1042/bj3340297 9716487 PMC1219691

[22] Ichihara M Hara T Kim H Murate T Miyajima A Oncostatin M and leukemia inhibitory factor do not use the same functional receptor in mice. Blood. (1997) 90:165–73. 9207450

[23] Levy JB Schindler C Raz R Levy DE Baron R Horowitz MC. Activation of the JAK-STAT signal transduction pathway by oncostatin-M cultured human and mouse osteoblastic cells. Endocrinology. (1996) 137:1159–65. 10.1210/endo.137.4.8625884 8625884

[24] Hermanns HM Radtke S Haan C Schmitz-Van de Leur H Tavernier J Heinrich PC. Contributions of leukemia inhibitory factor receptor and oncostatin M receptor to signal transduction in heterodimeric complexes with glycoprotein 130. J Immunol. (1999) 163:6651–8. 10586060

[25] Magrangeas F Boisteau O Denis S Jacques Y Minvielle S. Negative regulation of onconstatin M signaling by suppressor of cytokine signaling (SOCS-3). Eur Cytokine Netw. (2001) 12:309–15. 11399520

[26] Ehlting C Böhmer O Hahnel MJ Thomas M Zanger UM Gaestel M Oncostatin M regulates SOCS3 mRNA stability via the MEK–ERK1/2-pathway independent of p38MAPK/MK2. Cell Signal. (2015) 27:555–67. 10.1016/j.cellsig.2014.12.016 25562430

[27] Quintás-Cardama A Vaddi K Liu P Manshouri T Li J Scherle PA. Preclinical characterization of the selective JAK1/2 inhibitor INCB018424: therapeutic implications for the treatment of myeloproliferative neoplasms. Blood. (2010) 115:3109–17. 10.1182/blood-2009-04-214957 20130243 PMC3953826

[28] Verstovsek S Mesa RA Gotlib J Levy RS Gupta V DiPersio JF. A double-blind, placebo-controlled trial of ruxolitinib for myelofibrosis. N Engl J Med. (2012) 366:799–807. 10.1056/NEJMoa1110557 22375971 PMC4822164

[29] Allan EH Häusler KD Wei T Gooi JH Quinn JM Crimeen-Irwin B. EphrinB2 regulation by PTH and PTHrP revealed by molecular profiling in differentiating osteoblasts. J Bone Miner Res. (2008) 23:1170–81. 10.1359/jbmr.080324 18627264

[30] Allan EH Ho PWM Umezawa A Hata JI Makishia F Gillespie MT. Differentiation potential of a mouse bone marrow stromal cell line. J Cell Biochem. (2003) 90:158–69. 10.1002/jcb.10614 12938165

[31] Kaur S Raggatt LJ Millard SM Wu AC Batoon L Jacobsen RN. Self-repopulating recipient bone marrow resident macrophages promote long-term hematopoietic stem cell engraftment. Blood. (2018) 132:735–49. 10.1182/blood-2018-01-829663 29945953

[32] Joe AWB Yi L Natarajan A Le Grand F So L Wang J. Muscle injury activates resident fibro/adipogenic progenitors that facilitate myogenesis. Nat Cell Biol. (2010) 12:153. 10.1038/ncb2015 20081841 PMC4580288

[33] Mounier R Théret M Arnold L Cuvellier S Bultot L Göransson O. AMPKα1 regulates macrophage skewing at the time of resolution of inflammation during skeletal muscle regeneration. Cell Metab. (2013) 18:251–64. 10.1016/j.cmet.2013.06.017 23931756

[34] Banovac K Williams JM Patrick LD Haniff YM. Prevention of heterotopic ossification after spinal cord injury with indomethacin. Spinal Cord. (2001) 39:370–4. 10.1038/sj.sc.3101166 11464310

[35] Banovac K Williams JM Patrick LD Levi A. Prevention of heterotopic ossification after spinal cord injury with COX-2 selective inhibitor (rofecoxib). Spinal Cord. (2004) 42:707–10. 10.1038/sj.sc.3101628 15179440

[36] Sampath SC Sampath SC Ho ATV Corbel SY Millstone JD Lamb J. Induction of muscle stem cell quiescence by the secreted niche factor Oncostatin M. Nat Commun. (2018) 9:1531. 10.1038/s41467-018-03876-8 29670077 PMC5906564

[37] Tierney MT Aydogdu T Sala D Malecova B Gatto S Puri PL. STAT3 signaling controls satellite cell expansion and skeletal muscle repair. Nat Med. (2014) 20:1182–6. 10.1038/nm.3656 25194572 PMC4332844

[38] Price FD von Maltzahn J Bentzinger CF Dumont NA Yin H Chang NC. Inhibition of JAK-STAT signaling stimulates adult satellite cell function. Nat Med. (2014) 20:1174–81. 10.1038/nm.3655 25194569 PMC4191983

[39] Song HY Jeon ES Kim JI Jung JS Kim JH. Oncostatin M promotes osteogenesis and suppresses adipogenic differentiation of human adipose tissue-derived mesenchymal stem cells. J Cell Biochem. (2007) 101:1238–51. 10.1002/jcb.21245 17226768

[40] Sambasivan R Yao R Kissenpfennig A Van Wittenberghe L Paldi A Gayraud-Morel B. Pax7-expressing satellite cells are indispensable for adult skeletal muscle regeneration. Development. (2011) 138:3647–56. 10.1242/dev.067587 21828093

[41] Jenkins BJ Roberts AW Najdovska M Grail D Ernst M. The threshold of gp130-dependent STAT3 signaling is critical for normal regulation of hematopoiesis. Blood. (2005) 105:3512–20. 10.1182/blood-2004-09-3751 15650055

[42] Panopoulos AD Zhang L Snow JW Jones DM Smith AM El Kasmi KC. STAT3 governs distinct pathways in emergency granulopoiesis and mature neutrophils. Blood. (2006) 108:3682–90. 10.1182/blood-2006-02-003012 16888100 PMC1895456

[43] Mui AL Wakao H O'Farrell AM Harada N Miyajima A. Interleukin-3, granulocyte-macrophage colony stimulating factor and interleukin-5 transduce signals through two STAT5 homologs. Embo J. (1995) 14:1166–75. 10.1002/j.1460-2075.1995.tb07100.x 7720707 PMC398194

[44] Wright CR Ward AC Russell AP. Granulocyte colony-stimulating factor and its potential application for skeletal muscle repair and regeneration. Mediators Inflamm. (2017) 2017:7517350. 10.1155/2017/7517350 29362521 PMC5738577

[45] Pavey GJ Qureshi AT Tomasino AM Honnold CL Bishop DK Agarwal S. Targeted stimulation of retinoic acid receptor-γ mitigates the formation of heterotopic ossification in an established blast-related traumatic injury model. Bone. (2016) 90:159–67. 10.1016/j.bone.2016.06.014 27368930 PMC5546218

[46] Qureshi AT Dey D Sanders EM Seavey JG Tomasino AM Moss K. Inhibition of mammalian target of rapamycin signaling with rapamycin prevents trauma-induced heterotopic ossification. Am J Pathol. (2017) 187:2536–45. 10.1016/j.ajpath.2017.07.010 29029772 PMC5809339

[47] Cui M Ma X Sun J He J Shen L Li F. Effects of STAT3 inhibitors on neural functional recovery after spinal cord injury in rats. Biosci Trends. (2017) 10:460–6. 10.5582/bst.2016.01160 28003635

[48] Lacroix S Chang L Rose-John S Tuszynski MH. Delivery of hyper-interleukin-6 to the injured spinal cord increases neutrophil and macrophage infiltration and inhibits axonal growth. J Comp Neurol. (2002) 454:213–28. 10.1002/cne.10407 12442313

[49] Okada S Nakamura M Katoh H Miyao T Shimazaki T Ishii K. Conditional ablation of Stat3 or Socs3 discloses a dual role for reactive astrocytes after spinal cord injury. Nat Med. (2006) 12:829–34. 10.1038/nm1425 16783372

[50] Faulkner JR Herrmann JE Woo MJ Tansey KE Doan NB Sofroniew MV. Reactive astrocytes protect tissue and preserve function after spinal cord injury. J Neurosci. (2004) 24:2143–55. 10.1523/JNEUROSCI.3547-03.2004 14999065 PMC6730429

